# Investigating the Function of TaUBX57 in Enhancing Abiotic Stress Tolerance in Wheat

**DOI:** 10.3390/ijms26167995

**Published:** 2025-08-19

**Authors:** Min Jeong Hong, Chan Seop Ko, Man Bo Lee, Do Yoon Hyun, Dae Yeon Kim

**Affiliations:** 1 Advanced Radiation Technology Institute, Korea Atomic Energy Research Institute, 29 Geumgu, Jeongeup-si 56212, Jeollabuk-do, Republic of Korea; hongmj@kaeri.re.kr (M.J.H.); csko@kaeri.re.kr (C.S.K.); 2 Department of Plant Resources, Kongju National University, 54 Daehak-ro, Yesan-gun 32439, Chungcheongnam-do, Republic of Korea; manbolee@kongju.ac.kr; 3 Department of Crops and Forestry, Korea National University of Agriculture and Fisheries, 1515 Kongjwipatjwi-ro, Deokjin-gu, Jeonju-si 54874, Jeollabuk-do, Republic of Korea; 4 Research Center of Crop Breeding for Omics and Artificial Intelligence, Kongju National University, 54 Daehak-ro, Yesan-gun 32439, Chungcheongnam-do, Republic of Korea

**Keywords:** U-box protein, wheat, drought and salt stress, *TaUBX57*

## Abstract

Wheat (*Triticum aestivum* L.), a crucial global food crop, provides approximately 20% of daily protein and caloric intake globally. However, its production is increasingly threatened by abiotic stressors, especially drought and salinity, which are exacerbated by climate change. These stressors adversely affect plant growth, development, and yield, necessitating the development of stress-tolerant varieties. This study investigated the role of *TaUBX57*, a U-box E3 ubiquitin ligase, in enhancing stress tolerance in wheat. Functional domain analysis of TaUBX57 confirmed the presence of a conserved U-box and a protein kinase domain, suggesting its involvement in protein ubiquitination and stress responses. *TaUBX57*-overexpressing transgenic *Arabidopsis* lines exhibited significantly improved germination rates, root growth, and survival under drought and salt stress conditions. *TaUBX57* overexpression enhanced antioxidant enzyme activities and reduced the expression of oxidative stress markers, such as malondialdehyde. These findings highlight the potential role of *TaUBX57* in modulating stress-responsive pathways and enhancing abiotic stress tolerance, offering a promising avenue for developing more resilient wheat varieties through genetic engineering.

## 1. Introduction

Wheat (*Triticum aestivum* L.), one of the first domesticated food crops, has been a dietary staple for >8000 years, supporting major civilizations worldwide. Wheat plays a significant role in global food security and nutrition, providing ~20% of the daily intake of protein and calories. This essential grain meets the nutritional needs of billions. However, its production is increasingly challenged by biotic and abiotic stressors, such as climate change, diseases, and pests, which can negatively impact the quality and yield of wheat [1].

Climate change–induced drought and salinity stress pose significant threats to global food security by adversely affecting plant growth, development, and productivity, leading to reduced crop yields. Drought tolerance has emerged as a cost-effective strategy to enhance plant resilience to abiotic stressors. Concurrently, salt stress, exacerbated by natural and human-induced factors, severely impacts food crop production, particularly wheat, by causing ion toxicity and osmotic stress. Recent advancements in biotechnology, such as the identification and manipulation of stress-responsive genes, offer promising avenues for developing wheat varieties with enhanced tolerance to these stressors [2,3].

Ubiquitination is a crucial post-translational modification that regulates cellular functions, including protein degradation, DNA repair, and stress responses. It is orchestrated by a cascade of enzymatic activities involving E1 ubiquitin-activating enzymes, E2 ubiquitin-conjugating enzymes, and E3 ubiquitin ligases. E3 ligases are the most diverse and play a crucial role in recognizing specific substrates and facilitating the transfer of ubiquitin molecules from E2 enzymes to the substrates, thereby marking them for degradation by the 26S proteasome [4,5]. The ubiquitin–proteasome system is essential for maintaining cellular homeostasis and regulating cell cycle progression, signal transduction, stress responses, etc. [6,7]. Among the diverse classes of E3 ligases, the U-box-type is characterized by a conserved U-box domain, which is essential for ubiquitin ligase activity. U-box-type E3 ligases are involved in protein quality control, stress responses, etc. [8,9]. The U-box domain interacts with E2, facilitating the transfer of ubiquitin to target proteins. This interaction is crucial for the specificity and efficiency of ubiquitination, making U-box E3 ligases a critical component of the ubiquitin–proteasome system [10].

Plants have evolved intricate mechanisms to cope with abiotic stresses such as drought, salinity, and osmotic stress. Ubiquitination, mediated by E3 ligases, helps modulate the levels and activities of key stress-responsive proteins. This regulation is essential for plants to adapt and survive under adverse environmental conditions [11,12]. In addition to ubiquitination, plants deploy a complex antioxidant defense system to mitigate oxidative stress caused by abiotic stressors [11]. Key antioxidant enzymes include superoxide dismutase (SOD), catalase (CAT), and peroxidases (PODs) such as ascorbate peroxidase (APX) and glutathione peroxidase. These enzymes play a crucial role in scavenging reactive oxygen species (ROS) and maintaining cellular redox homeostasis [13]. The balance between ROS production and scavenging by antioxidant enzymes is critical for plant survival under stress, highlighting the importance of both ubiquitination and antioxidant defense mechanisms in plant stress responses.

In this study, functional domain analysis using InterProScan revealed that TaUBX57 contains a conserved U-box domain, indicating its potential role in ubiquitination. The domain structure suggests that TaUBX57 functions like other U-box E3 ligases, participating in protein quality control and stress responses. Phylogenetic analysis revealed that TaUBX57 shares a close evolutionary relationship with other U-box E3 ligases across plant species, suggesting conserved function. The evolutionary conservation of U-box E3 ligases across plant species underscores their fundamental role in stress responses and cellular homeostasis. Despite the known importance of E3 ligases in plant stress responses, the functions of TaUBX57 remain largely unexplored. This study aimed to elucidate the role of TaUBX57 in abiotic stress tolerance by investigating its function through overexpression in transgenic *Arabidopsis* plants. Specifically, we aimed to analyze the domain structure and evolutionary relationships of TaUBX57 to gain insights into its functional aspects. By determining the subcellular localization of TaUBX57, we aimed to understand its interaction with cellular components and its role in cellular processes. We evaluated the impact of *TaUBX57* overexpression on seed germination and root growth under osmotic, salt, and hormonal stress conditions to determine its role in stress tolerance. Additionally, we evaluated the physiological and biochemical responses of transgenic plants to drought and salt stress, focusing on parameters such as survival rates, growth performance, oxidative stress markers, and malondialdehyde (MDA) levels. Finally, we investigated the expression levels of antioxidant enzyme genes in transgenic plants under stress conditions, providing insights into how *TaUBX57* confers stress tolerance and regulates cellular responses to stress.

## 2. Results

### 2.1. Characterization of TaUBX57 as a U-Box E3 Ubiquitin Ligase in Wheat

**Figure 1:**
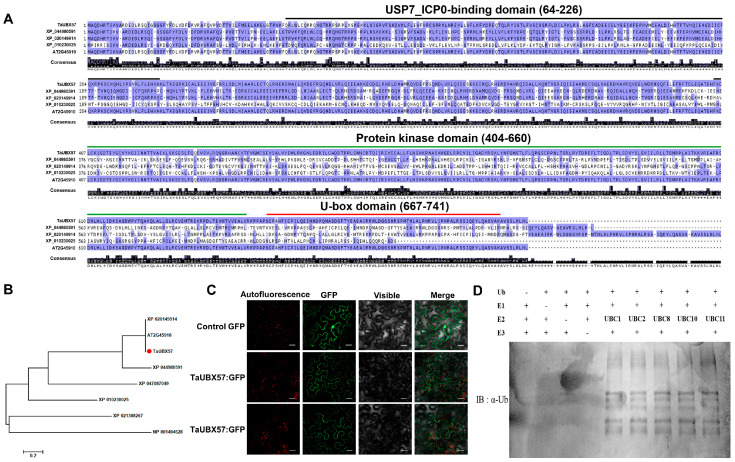
Domain and functional characterization of the wheat U-box domain protein TaUBX57. (**A**) Domain structure of TaUBX57 (TraesCS4D02G095000) was identified using InterProScan. The analysis revealed a conserved USP7_ICP0-binding domain at the N-terminal region (black line), U-box domain at the C-terminal region (amino acids 667–741, red line) and a protein kinase domain spanning amino acids 404–660 (green line). The domain structure was compared across several species, including XP_044980591 (*Hordeum vulgare*), XP_020149914 (*Aegilops tauschii*), and XP_010230025 (*Brachypodium distachyon*), highlighting conserved sequences indicative of similar functional roles. (**B**) A phylogenetic tree of TaUBX57 with other U-box proteins containing a protein kinase domain. The gene IDs included are XP_020149914 (*A. tauschii*), AT2G45910 (*Arabidopsis thaliana*), TaUBX57 (*Triticum aestivum*), XP_044980591 (*H. vulgare*), XP_047087049 (*Lolium rigidum*), XP_010230025 (*B. distachyon*), XP_021308267 (*Sorghum bicolor*), and NP_001404628 (*Oryza sativa japonica*). The analysis shows that TaUBX57 (red circle) is most closely related to XP_020149914 and AT2G45910, suggesting functional similarities in ubiquitin-mediated processes. (**C**) Subcellular localization of TaUBX57 fused with GFP in tobacco leaf epidermal cells. The TaUBX57-GFP fusion protein was transiently expressed under the CaMV 35S promoter. GFP fluorescence was predominantly observed along the plasma membrane. The red signal corresponds to the intrinsic autofluorescence of chloroplast. Confocal images were taken 72 h after infiltration. (**D**) In vitro ubiquitination assay demonstrating the E3 ligase activity of TaUBX57. Using recombinant TaUBX57 and E2 conjugating enzymes, polyubiquitin chains were formed, indicated by a smear of high-molecular-weight ubiquitinated proteins on the gel. This result confirms that TaUBX57 functions as an E3 ubiquitin ligase that can tag target proteins for degradation through the ubiquitin–proteasome system.

Domain analysis of TaUBX57 was conducted using InterProScan, revealing a conserved U-box domain at the C-terminal end (667–741 amino acids) and a protein kinase domain spanning amino acids 404–660 (Figure 1A). TaUBX57, identified as TraesCS4D02G095000 in common wheat (*Triticum aestivum*), shares structural features with similar proteins in other plant species, including XP_044980591 from *Hordeum vulgare*, XP_020149914 from *Aegilops tauschii*, and XP_010230025 from *Brachypodium distachyon*. This analysis indicated the presence of functionally significant domains in TaUBX57, likely involved in its activity as an E3 ubiquitin ligase and in kinase-related processes. The phylogenetic tree showed the evolutionary relationship of TaUBX57 with other U-box proteins containing a protein kinase domain (Figure 1B), including XP_020149914 (*A. tauschii*), AT2G45910 (*A. thaliana*), TaUBX57 (*T. aestivum*), XP_044980591 (*H. vulgare*), XP_047087049 (*Lolium rigidum*), XP_010230025 (*B. distachyon*), XP_021308267 (*Sorghum bicolor*), and NP_001404628 (*Oryza sativa japonica*). The analysis indicated that TaUBX57 is closely related to U-box proteins from *H. vulgare* (barley), *A. tauschii* (a wild relative of wheat), and *B. distachyon* (a model grass species). Notably, TaUBX57 exhibited the highest similarity with XP_020149914 from *A. tauschii* and AT2G45910 from *A. thaliana*. This finding supports the hypothesis that TaUBX57 shares functional similarities with these proteins, playing a similar role in ubiquitin-mediated protein degradation and stress response pathways. Analysis of subcellular localization, as depicted in the figure, revealed that TaUBX57 is predominantly localized to the plasma membrane. TaUBX57 localization was determined using a GFP fusion protein expressed in tobacco leaf epidermal cells. This finding suggests that TaUBX57 interacts with membrane-associated proteins or is involved in signaling pathways that are activated at the plasma membrane, particularly under stress conditions (Figure 1C). The results of the ubiquitination assay (Figure 1D) demonstrated that TaUBX57 functions as an E3 ubiquitin ligase. In vitro ubiquitination assays using recombinant TaUBX57 and E2 conjugating enzymes resulted in the formation of polyubiquitin chains, indicated by a smear of high-molecular-weight ubiquitinated proteins on the gel. These findings confirmed that TaUBX57 is an E3 ligase that facilitates the ubiquitination of target proteins.

### 2.2. Enhanced Germination and Early Growth in Arabidopsis Through TaUBX57 Overexpression

**Figure 2:**
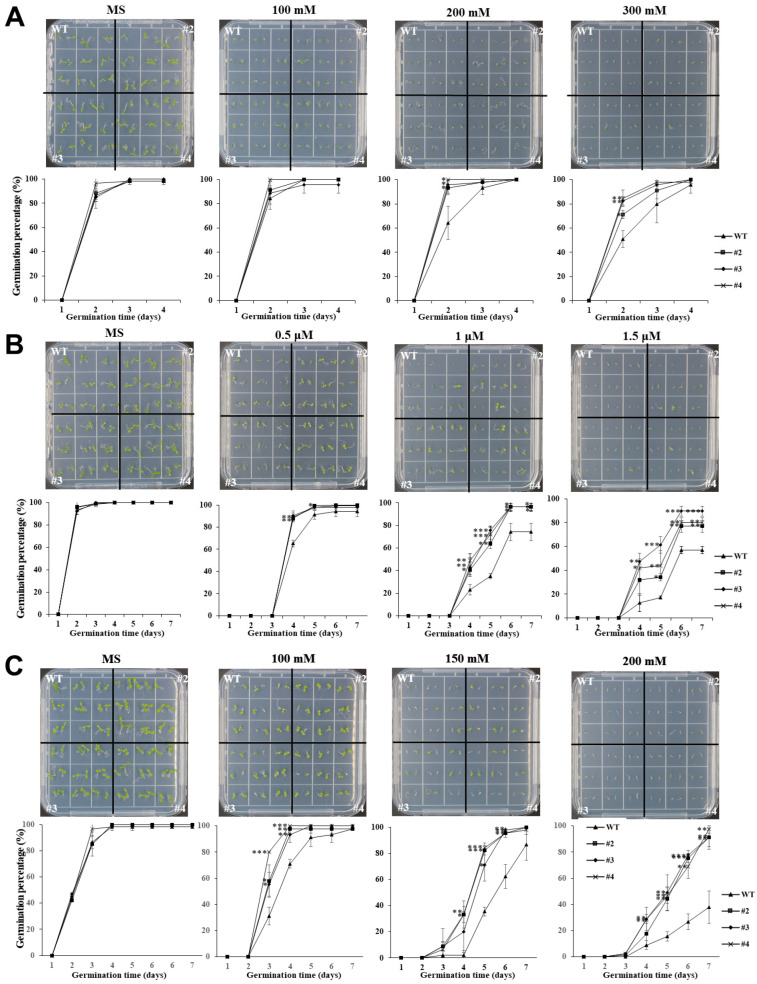
(**A**) Comparison of seed germination on 1/2 MS medium supplemented with 0, 100, 200, and 300 mM mannitol (**top**), and comparison of germination rates over 4 d (**bottom**). (**B**) Comparison of seed germination on 1/2 MS medium supplemented with 0, 0.5, 1.0, and 1.5 μM ABA (**top**), and comparison of germination rates over 7 d (**bottom**). (**C**) Comparison of seed germination on 1/2 MS medium supplemented with 0, 100, 150, and 200 mM NaCl (**top**), and comparison of germination rates over 7 d (**bottom**). All measured experiments were conducted with 50 replicates, and statistical analysis was performed using *t* tests. Error bars represent standard error (SE). Asterisks indicate levels of statistical significance compared with WT (* *p* < 0.05, ** *p* < 0.01, *** *p* < 0.001).

*TaUBX57* expression was analyzed using RT-qPCR under different stress conditions, including treatment with PEG, ABA, mannitol, and NaCl in wheat seedlings. *TaUBX57* expression increased across all treatments (Figure S1). Therefore, to investigate *TaUBX57*’s role in drought and salt stress tolerance, we developed transgenic *Arabidopsis* lines that overexpressed *TaUBX57*. The germination assay of *TaUBX57*-overexpressing transgenic *Arabidopsis* lines was assayed under various stress conditions to evaluate *TaUBX57*’s role in stress tolerance. The results showed significant differences (*p* value < 0.05) in germination rates and early seedling growth between *TaUBX57*-overexpressing lines and WT plants. The seeds were germinated on 1/2 MS medium supplemented with 0, 100, 200, and 300 mM mannitol. The *TaUBX57*-overexpressing lines (#2–4) demonstrated higher germination rates than WT plants, particularly at high mannitol concentrations (Figure 2A). This finding suggests that *TaUBX57* overexpression conferred increased tolerance to osmotic stress. Consistently, *TaUBX57*-overexpressing lines exhibited significantly higher germination rates than WT plants upon treatment with 0, 0.5, 1.0, and 1.5 µM ABA (Figure 2B). This finding suggests a role of *TaUBX57* in modulating ABA-related stress responses. The *TaUBX57*-overexpressing lines had higher germination rates than WT plants when seeds were exposed to different concentrations of NaCl (0, 100, 150, and 200 mM), even under high-salinity conditions (Figure 2C). Overall, these results suggest that *TaUBX57* overexpression increased germination and early seedling growth under osmotic, ABA, and salt stress conditions, indicating a role for *TaUBX57* in improving stress resilience during the early stages of seedling development (Figure 2).

### 2.3. Enhanced Root Growth in TaUBX57-Overexpressing Arabidopsis Lines

**Figure 3:**
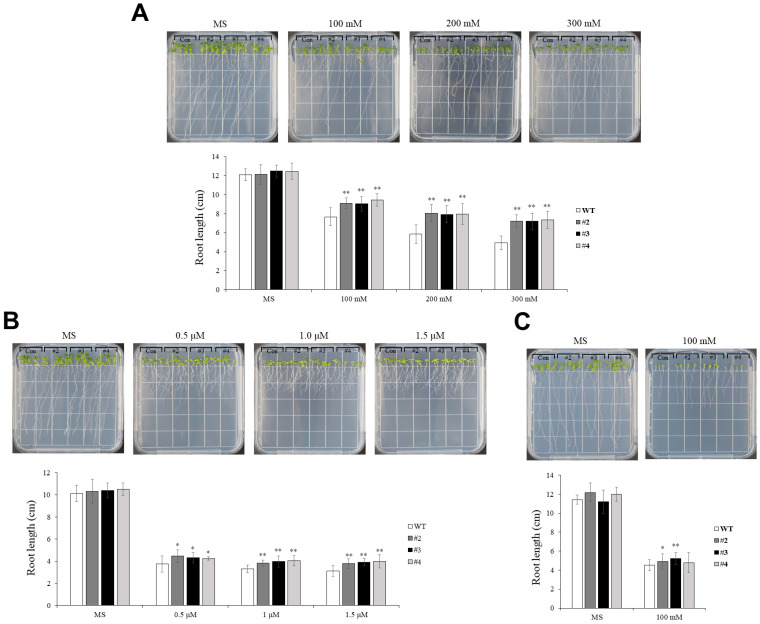
Four-day-old seedlings were transferred to 1/2 MS medium with various concentrations of (**A**) mannitol, (**B**) ABA, and (**C**) NaCl. Status of root growth (**top**) and comparison of root lengths after 7 d (**bottom**). The images show root development in wild-type (WT) plants and *TaUBX57*-overexpressing lines (#2–4) under stress, highlighting differences in root elongation between the genotypes. All experiments were conducted with 10 replicates, and statistical analysis was performed using *t* tests. Error bars represent standard error (SE). Asterisks indicate levels of statistical significance compared with WT (* *p* < 0.05, ** *p* < 0.01).

Root growth in WT and *TaUBX57*-overexpressing *Arabidopsis* lines (#2–4) was evaluated on 1/2 MS medium supplemented with 0, 100, 200, and 300 mM mannitol. The images show root length and density after 7 d of treatment (Figure 3A). Quantification of average root length indicated that the *TaUBX57*-overexpressing lines had longer roots than WT plants, especially under higher concentrations of mannitol, suggesting enhanced tolerance to osmotic stress. Similarly, WT and *TaUBX57*-overexpressing lines were grown on 1/2 MS medium with 0, 0.5, 1.0, and 1.5 µM ABA. Root growth was observed after 7 d (Figure 3B). Quantification of average root length revealed that the *TaUBX57*-overexpressing lines had significantly longer roots than WT plants under ABA treatment, indicating a potential role of *TaUBX57* in modulating ABA-related growth inhibition. Root growth was assessed on 1/2 MS medium supplemented with varying concentrations of NaCl (0, 100, 150, and 200 mM). Root growth and length were quantified after 7 d (Figure 3C). The *TaUBX57*-overexpressing lines exhibited longer roots than WT plants, particularly at higher NaCl concentrations, indicating that *TaUBX57* increased tolerance to salt stress in *Arabidopsis*.

### 2.4. Enhanced Stress Tolerance in TaUBX57-Overexpressing Arabidopsis Lines

**Figure 4:**
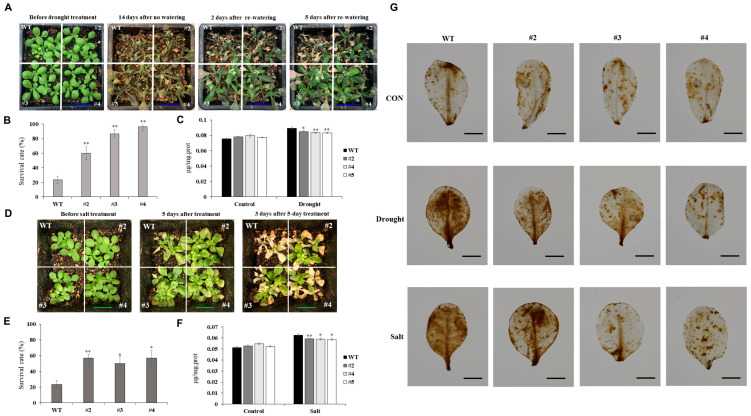
Phenotypic and physiological responses of control (wild-type [WT]) and transgenic *Arabidopsis* plants under drought and salt stress conditions. (**A**) Comparison of growth status between control (wild-type [WT]) and transgenic *Arabidopsis* plants (#2–4) at 2 and 5 d after rewatering, following 14 d of no watering (drought stress). Scale bar = 0.25 cm (blue). (**B**) Survival rate of WT and transgenic lines under drought stress. (**C**) Comparison of malondialdehyde (MDA) content, an indicator of lipid peroxidation and oxidative stress, before and after drought treatment. (**D**) Comparison of growth status between control and transgenic plants before NaCl treatment, after treatment with 300 mM NaCl for 7 d, and 3 d after the 7 d treatment. Scale bar = 0.2 cm (green). (**E**) Survival rate under salt stress. (**F**) Comparison of MDA content before and after salt treatment. (**G**) DAB staining assay for detecting damage due to reactive oxygen species (ROS) under control, drought, and salt stress conditions. Scale bar = 0.5 cm (black). All experiments were conducted with 12 replicates, and statistical analysis was performed using *t* tests. Error bars represent standard error (SE). Asterisks indicate levels of statistical significance compared with WT (* *p* < 0.05, ** *p* < 0.01).

The growth status of the plants was monitored at three key stages: before drought treatment, 14 d after withholding water, and 2 and 5 d after rewatering (Figure 4A). Under drought conditions, the *TaUBX57*-overexpressing lines had a higher survival rate than WT plants (Figure 4B). The MDA content, an indicator of lipid peroxidation and oxidative stress, was measured before and after drought treatment (Figure 4C). The results indicated that the *TaUBX57*-overexpressing lines had lower MDA content than WT plants, suggesting reduced oxidative damage.

For the salt stress experiment, plants were exposed to 300 mM NaCl for 7 d, and growth status was assessed before treatment, immediately after treatment, and 3 d after treatment (Figure 4D). Under salt stress, the *TaUBX57*-overexpressing lines had a higher survival rate than WT plants (Figure 4E). Furthermore, the MDA content before and after salt treatment was measured (Figure 4F), indicating that the *TaUBX57*-overexpressing lines experienced less oxidative stress than WT plants. DAB staining was used to detect ROS damage under control, drought, and salt stress conditions (Figure 4G).

Under both drought and salt stress conditions, the *TaUBX57*-overexpressing lines displayed less intense staining than WT plants, indicating lower ROS levels. Overall, these results suggest that *TaUBX57* overexpression increased tolerance to drought and salt stress in *Arabidopsis*, potentially by reducing oxidative damage and improving survival rates (Figure 4).

### 2.5. Enhanced Antioxidant Activity and Stress Tolerance in TaUBX57-Overexpressing Arabidopsis Lines

**Figure 5:**
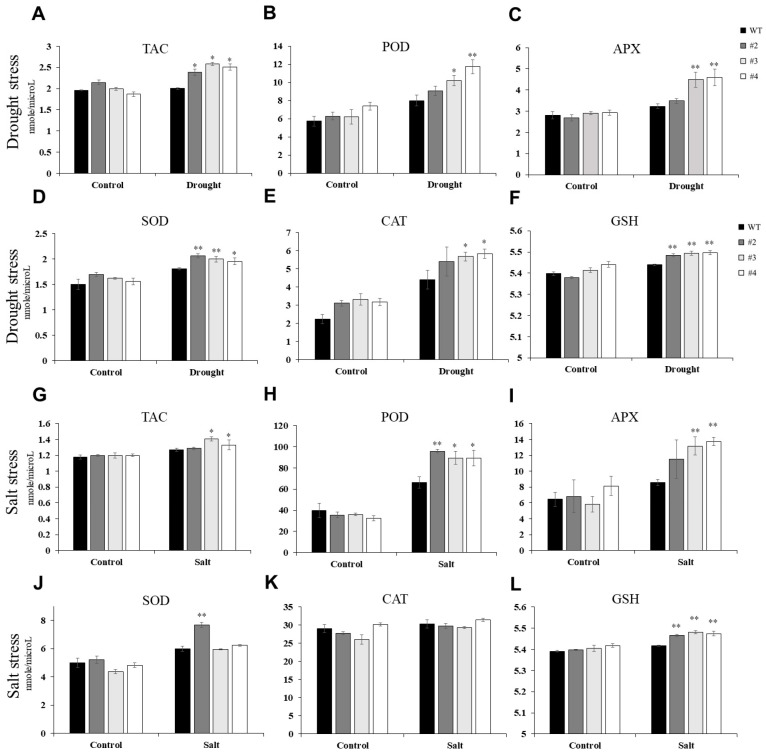
Antioxidant enzyme activity assays in *TaUBX57*-overexpressing *Arabidopsis* lines under drought (**top** panels) and salt (**bottom** panels) stress conditions. (**A**,**G**) Total antioxidant capacity (TAC) and the activities of (**B**,**H**) peroxidase (POD), (**C**,**I**) ascorbate peroxidase (APX), (**D**,**J**) superoxide dismutase (SOD), (**E**,**K**) catalase (CAT), and (**F**,**L**) glutathione (GSH) were measured. Each experiment was conducted with three biological replicates. Statistical significance was assessed using *t* tests, with error bars representing standard error of the mean (SE). Asterisks indicate significant differences between overexpressing lines and wild-type (WT) plants (* *p* < 0.05, ** *p* < 0.01).

In *Arabidopsis*, *TaUBX57* overexpression increased tolerance to drought and salt stress, potentially by reducing oxidative damage and improving survival rates under adverse conditions (Figure 4). Measurement of TAC and the activities of POD, APX, SOD, and CAT showed that *TaUBX57*-overexpressing lines exhibited significantly higher levels of TAC than WT plants, especially under stress conditions (Figure 5). Under drought stress, POD and APX activities were significantly increased in two *TaUBX57*-overexpressing lines (Figure 5B,C), whereas SOD and glutathione (GSH) activities were significantly elevated in all three *TaUBX57*-overexpressing lines (Figure 5D,F). Under salt stress, POD activity significantly increased in all three *TaUBX57*-overexpressing lines (Figure 5H), suggesting enhanced detoxification of ROS under saline conditions. SOD activity was significantly upregulated in one of the *TaUBX57*-overexpressing lines (Figure 5J), further supporting *TaUBX57*’s role in enhancing antioxidant defense. GSH activity significantly increased in all three *TaUBX57*-overexpressing lines (Figure 5I). This enhanced antioxidant capacity in *TaUBX57*-overexpressing lines suggests a robust mechanism for mitigating oxidative stress, thereby contributing to the improved stress tolerance in these plants.

## 3. Discussion

Overall, we analyzed TaUBX57 and confirmed its identity as a U-box-type E3 ubiquitin ligase (Figure 1). Domain structure analysis and phylogenetic analysis indicated conserved function across species (Figure 1A). Analysis of subcellular localization and the results of the ubiquitination assay suggested its role in stress response pathways (Figure 1C). These findings lay the groundwork for further investigations into the specific substrates and pathways regulated by TaUBX57 in wheat and related species (Figure 1D).

The germination assay of transgenic *Arabidopsis* overexpressing *TaUBX57* revealed enhanced stress tolerance under various conditions. The *TaUBX57*-overexpressing lines (#2–4) exhibited significantly higher germination rates than WT plants across all treatments, including osmotic stress induced by mannitol, ABA, and salt stress (Figure 2). Specifically, these transgenic lines showed improved germination at higher mannitol concentrations (0, 100, 200, and 300 mM), increased tolerance under ABA (0, 0.5, 1.0, and 1.5 µM), and better performance under NaCl-induced salt stress (0, 100, 150, and 200 mM). Correspondingly, the accompanying bar graphs quantifying average root length indicate that the *TaUBX57*-overexpressing lines had longer roots than WT plants across all treatments (Figure 3). Under higher concentrations of mannitol, the *TaUBX57*-overexpressing lines exhibited longer roots, suggesting enhanced tolerance to osmotic stress (Figure 3A). Under ABA treatment conditions, the *TaUBX57*-overexpressing lines showed significantly longer roots than WT plants, indicating that *TaUBX57* can modulate ABA-related growth inhibition (Figure 3B). Under salt stress conditions, the *TaUBX57*-overexpressing lines displayed longer roots than WT plants, particularly at higher NaCl concentrations, demonstrating that *TaUBX57* enhanced tolerance to salt stress (Figure 3C). These results suggest that *TaUBX57* overexpression may enhance stress tolerance by supporting cellular water balance, modulating ABA signaling pathways, and managing ion homeostasis, thereby facilitating germination and enhancing stress tolerance at the seedling stage.

U-box E3 ligases played significant roles in managing plant responses to abiotic stressors, including drought, salt stress, and ABA. For instance, in rice, the U-box E3 ubiquitin ligase *OsPUB67* enhanced drought tolerance by promoting ROS scavenging and facilitating stomatal closure, a mechanism crucial for managing water loss and oxidative stress under drought conditions [14]. Similarly, in apple, MdPUB29 positively regulated salt stress tolerance, likely by maintaining cellular ion balance and osmotic regulation. *MdPUB29* expression was significantly reduced under salt stress, highlighting its role in salt tolerance [15]. Conversely, in soybean, *GmPUB21* negatively regulated the stress response to drought and salinity. The overexpression of GmPUB21 increased sensitivity to these stressors, characterized by higher ROS accumulation and altered stomatal behavior. However, silencing *GmPUB21* enhanced stress tolerance, suggesting that it modulated the stress response through ABA signaling [16]. This dual role of GmPUB21 highlights the complex regulatory mechanisms that U-box E3 ligases can exert in different plants and stress conditions.

Poplar *PalPUB79* positively regulated ABA signaling and enhanced drought tolerance. *PalPUB79* expression was associated with increased ABA sensitivity, which helped in regulating the osmotic stress response under drought conditions [17]. Moreover, a comprehensive study on sorghum identified multiple U-box genes involved in salt stress responses, indicating their significant role in enhancing salinity tolerance and potentially contributing to improved crop resilience [18]. In Arabidopsis, U-box E3 ligases, such as AtPUB18, AtPUB19, and AtPUB44, negatively regulated seed germination under salt stress and ABA signaling, further demonstrating the diverse regulatory roles of these ligases [19]. Recent research highlighted the role of U-box E3 ligases in modulating ABA signaling, with studies demonstrating their involvement in fine-tuning the response to ABA [20].

Although ROS are byproducts of normal cellular metabolism, they can accumulate to harmful levels under abiotic stressors, like drought and salinity, leading to the oxidative damage of proteins, lipids, and nucleic acids. Plants counteract this by activating a complex antioxidant defense system, including enzymatic (SOD, CAT, and POD) and nonenzymatic (ascorbate and GSH) components [13,21,22]. These systems work together to scavenge excessive ROS, maintaining cellular redox balance and protecting plants from oxidative stress. Drought stress can exacerbate ROS production due to water deficit, whereas salt stress leads to osmotic stress and ionic toxicity, both resulting in increased ROS levels. The role of antioxidants is crucial in ROS detoxification, preventing cellular damage and ensuring plant survival under these conditions [13,21]. In our study, transgenic *Arabidopsis* lines overexpressing *TaUBX57* demonstrated significantly enhanced antioxidant enzyme activities under both drought and salt stresses. The measured enzymes, including TAC, POD, APX, SOD, CAT, and GSH, were upregulated in these lines compared with WT plants, suggesting an enhanced capacity to detoxify ROS and protect cellular structures from oxidative damage (Figure 5). Consequently, MDA content was lower in TaUBX57-overexpressing plants than in controls under both drought and salt stress conditions (Figure 4C, F). Consistently, ROS damage, assessed through DAB staining, was lower in TaUBX57-overexpressing plants than in controls, further supporting high antioxidant defense in transgenic lines (Figure 4G). This reduction in MDA and ROS damage highlights the role of TaUBX57 in mitigating oxidative stress, thereby contributing to the overall stress tolerance in these plants.

This study highlights the crucial role of U-box E3 ligases, specifically TaUBX57, in enhancing stress tolerance in plants. The findings underscore the potential of these proteins to enhance or mitigate stress responses, depending on the specific pathway or mechanism involved. These insights are invaluable for developing crops with improved tolerance to environmental stresses, especially as climate change increases the frequency and severity of abiotic stresses such as drought and salinity. Understanding the role of TaUBX57 in stress responses not only enriches our knowledge of the mechanisms of stress tolerance in wheat but also offers potential genetic targets for developing stress-tolerant crops. The ability to engineer crops that can better withstand environmental challenges is critical for ensuring agricultural sustainability and food security in a changing climate. This study provides a foundation for biotechnological applications aimed at enhancing crop resilience through genetic engineering and breeding programs.

Although the role of *TaUBX57* in enhancing stress tolerance through increased antioxidant activity has been established, the specific protein substrates targeted for degradation by *TaUBX57* remain unidentified. Identifying these substrates is crucial for understanding how *TaUBX57* confers stress tolerance. Studies could employ proteomics approaches, such as yeast two-hybrid assays, to screen for interacting partners of TaUBX57. The findings of this study will be pivotal in elucidating the functional landscape of TaUBX57 in plant stress responses and could lead to the development of more resilient crop varieties.

## 4. Materials and Methods

### 4.1. Plant Materials and Stress Treatments

Colored wheat (K4191) of the F4:8 generation from the cross between “Woori-mil” (National Agrobiodiversity Center, RDA, Republic of Korea; accession no. IT172221) and “D-7” (an inbred line developed by Korea University; Fleming4/3/PIO2580//T831032/Hamlet) varieties was utilized as plant material [23]. Wheat seeds were soaked in water until rooting and germination and transferred to half-strength Hoagland’s solution. The hydroponic culture system was maintained using Hoagland’s solution adjusted to pH 5.5–6.5, which was freshly prepared and renewed on a daily basis to maintain nutrient consistency and minimize microbial growth. The seedlings were grown for 10 d at 22 °C with a 16 h light/8 h dark photoperiod. To induce drought stress, seedlings were transferred to Hoagland’s solution containing 20% PEG-6000. For abscisic acid (ABA) treatment, seedlings were transferred to 100 μM ABA. For salt stress, 200 mM NaCl was used. The seedlings were exposed for 36 h before sampling.

### 4.2. Gene Cloning and Sequence Analysis

The open reading frame sequence of *TaUBX57* was obtained from Ensembl Plants (https://plants.ensembl.org/Triticum_aestivum/Info/Index; accessed on 2 February 2023). Homologous genes in other species were identified using the NCBI database (www.ncbi.nlm.nih.gov; accessed on 2 February 2023) and BLASTn tool. Significant sequences were downloaded, and domain prediction was performed using InterProScan (https://www.ebi.ac.uk/interpro/search/sequence; accessed on 2 February 2023). Multiple sequence alignments were performed using ClustalW [24], and a phylogenetic tree was constructed using the neighbor-joining method in MEGA7 software (version 7.0; MEGA Software, Pennsylvania State University, University Park, PA, USA).

### 4.3. In Vitro Ubiquitination Assay and Western Blotting

The full-length coding sequence of *TaUBX57* was cloned into pDEST-HisMBP (Plasmid #11085, Addgene, Cambridge, MA, USA) using LR Clonase (Thermo Fisher Scientific, Waltham, MA, USA). MBP-tagged TaUBX57 was expressed in *E. coli* BL21 (DE3) pLysS and purified using amylose resin (New England BioLabs, Ipswich, MA, USA). The in vitro ubiquitination assay was performed as described by Choo and Zhang [25] and Zeng [26], with modifications. The assay was performed in a total reaction volume of 50 μL, containing 1 μg of purified MBP-TaUBX57, 10 μg of ubiquitin (Sigma, St. Louis, MO, USA), 40 ng of E1 (Boston Biochem, Cambridge, MA, USA), and 100 ng of various E2 enzymes. The reactions were incubated at 30 °C for 3 h, stopped with SDS sample buffer, and heated at 100 °C for 5 min. Ubiquitinated substrates were separated using 12% SDS-PAGE and identified via Western blotting using an anti-ubiquitin antibody (Sigma, MO, USA). The signals were detected using an ECL kit (Merck, Rahway, NJ, USA) and iBright CL1000 Imaging System (Thermo Scientific).

### 4.4. Subcellular Localization

For subcellular localization, full-length *TaUBX57* was cloned into the pMDC43 vector containing GFP using the Gateway method [27]. The constructs pMDC43 and pMDC43 (control) were introduced into *Agrobacterium* strain GV3101 and infiltrated into the abaxial surface of *Nicotiana benthamiana* leaves. Fluorescence was detected 72 h post-infiltration using a confocal laser-scanning microscope (Zeiss LSM800, Oberkochen, Germany). Green fluorescence from GFP was visualized using excitation at 488 nm, with signal detection in the 510–530 nm emission range. Red autofluorescence from chloroplasts was observed by applying a 633 nm excitation laser and collecting emitted light above 650 nm.

### 4.5. RNA Extraction and Gene Expression

Total RNA was extracted from wheat seedlings using TRIzol reagent (Invitrogen, Waltham, MA, USA) and treated with DNase I to remove residual DNA. First-strand cDNA was synthesized from 1 μg of total RNA using a Power cDNA Synthesis Kit (iNtRON Biotechnology, Gyeonggi-do, Republic of Korea). RT-qPCR was performed using 2× SYBR Green Premix Ex Taq II (Takara, Kusatsu, Shiga, Japan) on a Bio-Rad Opus 96 System with the following parameters: 95 °C for 5 min followed by 45 cycles of 95 °C for 10 s and 65 °C for 30 s. Actin (AB18991) was used as an internal reference. Experiments were conducted with three biological replicates, and gene expression variations were evaluated using the 2^−ΔΔCt^ method [28]. Specific primers are listed in Table S1.

### 4.6. Arabidopsis Transformation and Stress Treatments

*Arabidopsis thaliana* ecotype Landsberg *erecta* (Ler-0) was used as the wild type (WT). The full-length coding region of *TaUBX57* was cloned into pMDC32 and transformed into *Agrobacterium* GV3101 for the transformation of *Arabidopsis* using the floral dip method [29]. Transgenic seeds were selected on 1/2 MS medium with 50 μg/mL hygromycin. Three independent homozygous T_3_ transgenic lines (#2–4) were selected based on phenotypic consistency and elevated TaUBX57 transcript levels, as verified by RT-qPCR analysis (Figure S2). For stress treatment, seedlings were transferred to soil and grown at 22 °C under long-day conditions (16 h light/8 h dark). Drought stress was induced by withholding water for 14 d, and salt stress was applied using 300 mM NaCl. Plant phenotypes were monitored during stress and survival and assessed after 5 d of rehydration.

### 4.7. Arabidopsis Seed Germination and Root Growth Assay

Sterilized seeds were sown on 1/2 MS medium supplemented with 0, 100, 200, and 300 mM mannitol; 0, 0.5, 1.0, and 1.5 μM ABA; or 0, 100, 150, and 200 mM NaCl. Germination rates were assessed daily over 7 d, noting radicle emergence and cotyledon greening. For root growth assays, 4-day-old seedlings were transferred to 1/2 MS medium with various concentrations of mannitol, ABA, and NaCl, and root lengths were measured after 7 d.

### 4.8. Antioxidant Activity Assays

Crude enzyme extracts were prepared from 100 mg of *Arabidopsis* tissue using a protein extraction solution containing 50 mM potassium phosphate buffer (pH 7.5). The activities of APX, CAT, POD, SOD, and total antioxidant capacity (TAC) were measured using commercial kits: APX (MBS2548460, MyBiosource, San Diego, CA, USA), CAT (MBS8243260, MyBiosource, CA, USA), POD (KTB1150, Abbkine, Wuhan, China), SOD (WST-1 method, MBS2540402, MyBiosource, CA, USA), and TAC (MAK187, Sigma, MO, USA). Protocols and calculations followed the manufacturers’ instructions.

### 4.9. MDA Content

MDA content was measured using a kit (MBS2540407, MyBiosource, San Diego, CA, USA). Approximately 0.1 g of *Arabidopsis* tissue was ground and extracted, followed by analysis according to the assay protocol.

### 4.10. 3,3′-Diaminobenzidine (DAB) Assay

ROS damage was visualized using DAB staining [30]. Rosette leaves which had been subjected to drought and salt stress treatments for 5 d were immersed in 1% DAB solution until brown spots appeared, before being treated with destaining solution (acetic acid, glycerol, and 96% ethanol in a 1:1:3 ratio) and boiled. After destaining was complete, the leaves were photographed in water.

### 4.11. Statistical Analysis

Statistical significance was determined using SPSS software (version 25.0) using *t* tests. Significant differences are indicated by asterisks above columns in the figures (* *p* < 0.05, ** *p* < 0.01).

## 5. Conclusions

This study demonstrates the critical role of the U-box E3 ubiquitin ligase TaUBX57 in enhancing plant tolerance to abiotic stresses, specifically for drought and salinity. *TaUBX57* overexpression in transgenic *Arabidopsis* significantly improved stress-related traits, including higher germination rates and longer root lengths under various stress conditions. These improvements are attributed to the increased antioxidant activity and the modulation of ABA signaling pathways, which collectively mitigate oxidative damage and maintain cellular homeostasis. The observed reduction in ROS accumulation and MDA content in the *TaUBX57*-overexpressing lines further underscores the ligase’s protective role against oxidative stress.

Our findings emphasize the potential of *TaUBX57* as a promising genetic target for the development of stress-tolerant crop varieties—an increasingly important goal given the increasing incidence of abiotic stresses driven by climate change. Although further research is necessary to identify the specific protein substrates targeted by *TaUBX57* to fully elucidate its role in stress response pathways, the findings of this study provide a strong foundation for future biotechnological applications. By harnessing the stress tolerance conferred by *TaUBX57*, we could engineer crops with enhanced resilience to environmental challenges, thereby supporting agricultural sustainability and food security. This study reinforces the importance of U-box E3 ligases in plant stress responses and their potential as targets for genetic improvement in crop-breeding programs.

## Data Availability

All data relevant to this study are included within the article and its Supplementary Files.

## Supplementary Materials

The following supporting information can be downloaded at: https://www.mdpi.com/article/10.3390/ijms26167995/s1.

## Abbreviations

The following abbreviations are used in this manuscript:

SOD	Superoxide dismutase
CAT	Catalase
POD	Peroxidase
APX	Ascorbate peroxidase
ROS	Reactive oxygen species
MDA	Malondialdehyde
ABA	Abscisic acid
TAC	Total antioxidant capacity
DAB	3,3′-Diaminobenzidine
GSH	Glutathione
WT	Wild-type
SE	Standard error
