# Supplementary figures and images for: Investigating the Function of TaUBX57 in Enhancing Abiotic Stress Tolerance in Wheat
Source: Int J Mol Sci. 2025 Aug 19;26(16):7995. doi: 10.3390/ijms26167995 (PMC12386796; doi:10.3390/ijms26167995)

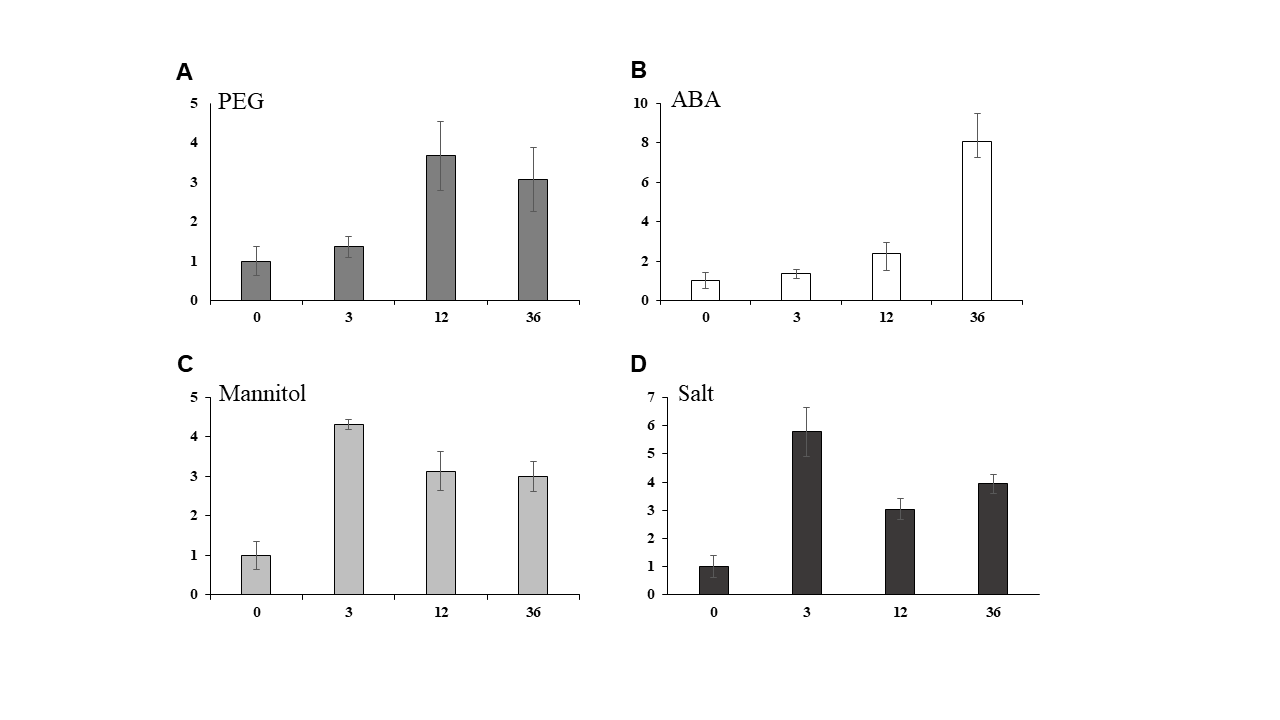

Supplement: Supplementary file 1 [file ijms-26-07995-s001.zip › Supple_IJMS/Figure_S1.TIF]

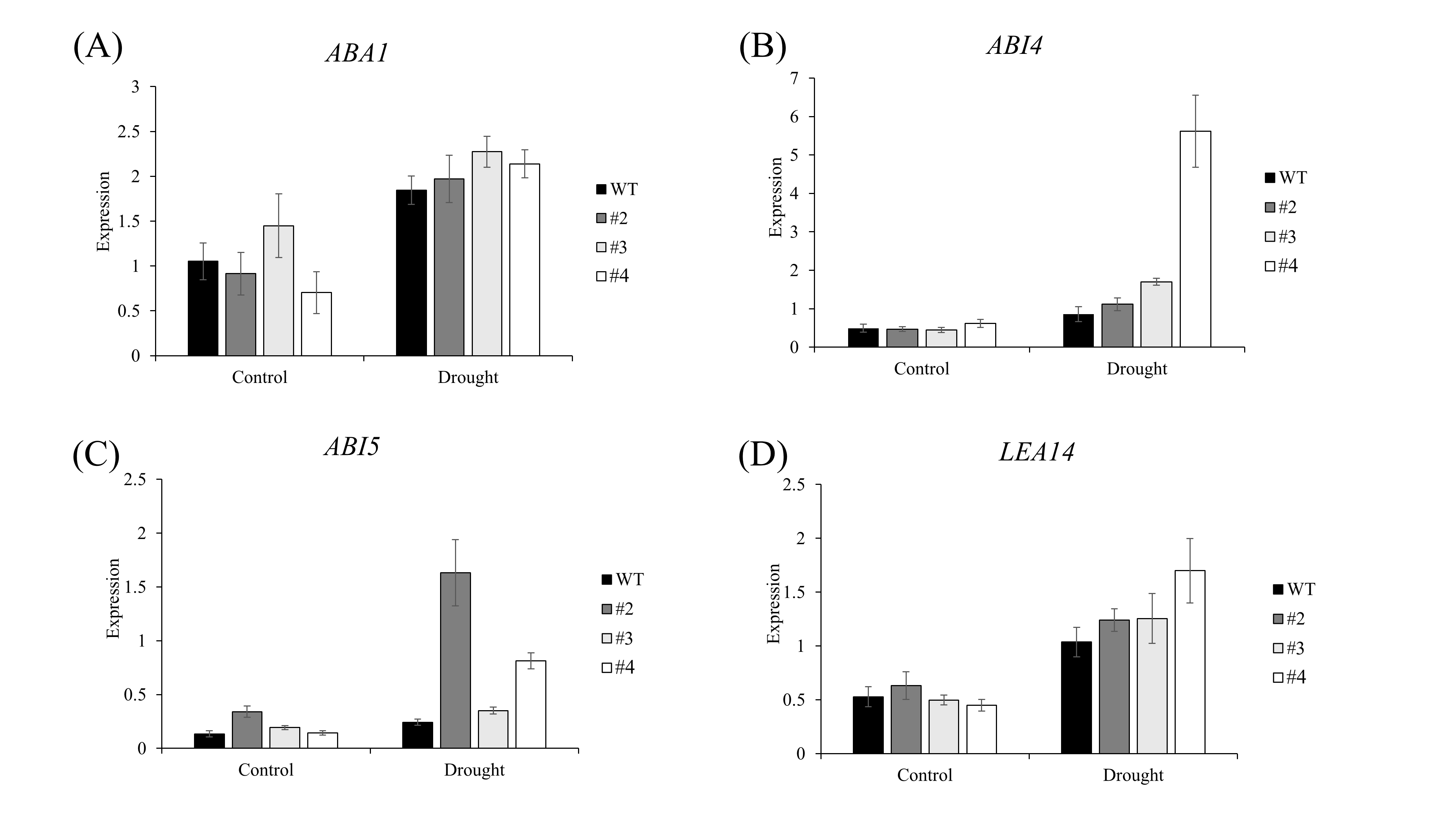

Supplement: Supplementary file 1 [file ijms-26-07995-s001.zip › Supple_IJMS/Figure_S2.tif]

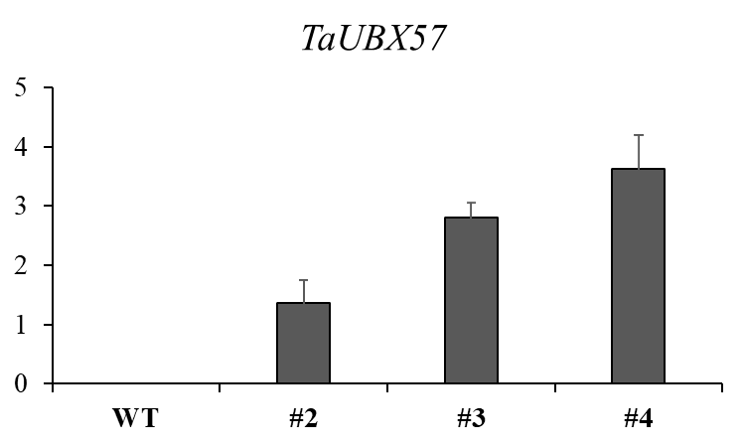

Supplement: Supplementary file 1 [file ijms-26-07995-s001.zip › Supple_IJMS/Figure_S3.tif]
